# Adult Type Granulosa Cell Tumor: A Very Rare Case of Sex-Cord Tumor of the Testis with Review of the Literature

**DOI:** 10.1155/2013/932086

**Published:** 2013-05-16

**Authors:** Dimosthenis Miliaras, Eleftherios Anagnostou, Ioannis Moysides

**Affiliations:** ^1^Laboratory of Histology and Embryology, Faculty of Medicine, Aristotle University of Thessaloniki, 54006 Thessaloniki, Greece; ^2^Department of Pathology, Euromedica Geniki Kliniki of Thessaloniki, 2 Gravias Street, 54645 Thessaloniki, Greece

## Abstract

Granulosa cell tumor (GST) is a sex-cord/stromal neoplasm of the gonads, more commonly arising in the ovaries, while approximately 80 cases have been reported in the testes. Out of these, 30 cases were of the adult type, while the remainder 50 cases were of the juvenile type. The latter mostly concerned infants and followed a benign course. However, the adult type testicular GCTs may be potentially malignant as it also happens in female patients with such neoplasms. We present a case of an adult type GCT located at the left testis. The patient was subjected to total orchiectomy and received no further treatment. Histology showed typical GCT histomorphology with Call-Exner bodies in some places. The immunoprofile of the tumor was CD99 (+), calretinin (+), inhibin (+), alpha smooth muscle actin (+), vimentin (+), ER (−), PR (−), keratin AE1/AE3 (−), alpha fetoprotein (−), CD117 (−), and placental alkaline phosphatase (−). Two years after surgery, the patient is alive and well with no signs of recurrence.

## 1. Introduction

Granulosa cell tumor (GST) belongs to the sex-cord/stromal tumors of the gonads. Two forms of GST have been recognized, namely, the typical adult type, and its variation, and the juvenile type. GST arises far more commonly in the ovaries but on rare occasions may be encountered in the testes. Most of the latter cases concerned infants, which presented the juvenile type morphology [1–3], while the minority was adult type GSTs [4–10]. In this paper, we describe a case of typical adult type GST arising in the testis of an adult male.

## 2. Case Presentation

**Figure 1:**
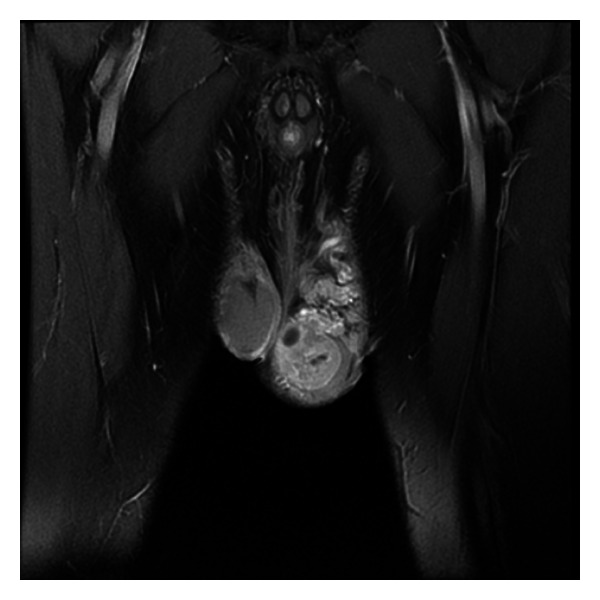
Coronal T1-weighted MRI image demonstrating a tumor that occupies the largest part of the left testis.

 A 37-year-old white male attended our hospital for scrotal swelling. He did not present any signs and symptoms suggestive of a hormone-secreting tumor like gynecomastia, and his history was otherwise unremarkable. Palpation, ultrasound examination, and MRI revealed a tumor of the left testis (Figure 1), and so surgical excision was decided. His preoperative blood tests were within normal limits, and the abdominal and thoracic CT scans did not show any significant lymph node enlargement or distant metastases. The patient was operated upon, and a left total orchiectomy was performed. His postoperational course was uneventful. The patient was released from the hospital with no further treatment, and he is being followed at six-month intervals for two years now. No signs of recurrence or metastasis have been identified yet.

## 3. Pathological Findings

**Figure 2:**
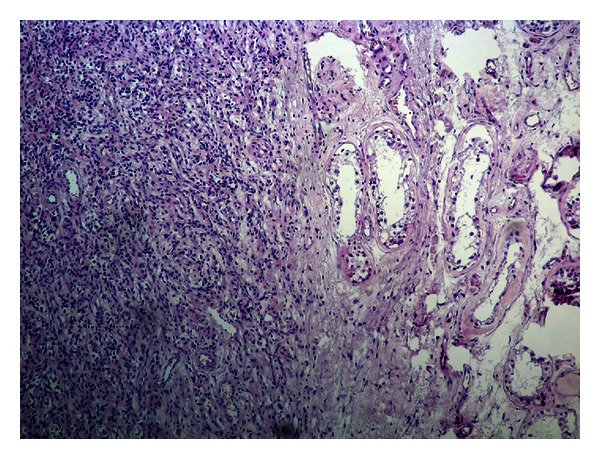
Granulosa tumor of the testis with trabecular and diffuse solid pattern is seen on the left part of the picture, while atrophic testicular parenchyma is seen on the right part of the picture (H&E ×100).

**Figure 3:**
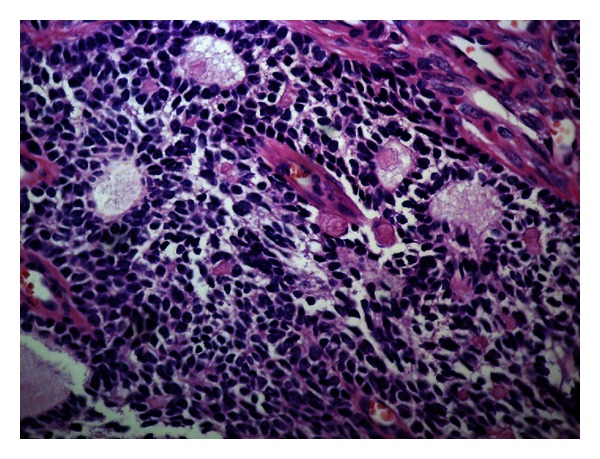
The tumor shows an area with many characteristic Call-Exner bodies (H&E ×400).

**Figure 4:**
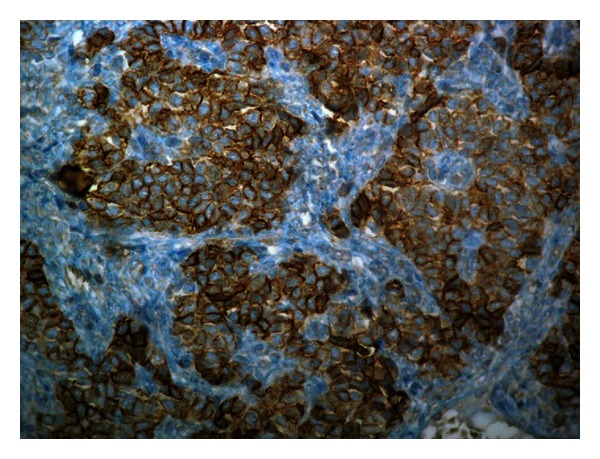
Tumor cells present strong membrane reaction to CD99 (DAB, Haematoxylin ×400).

**Figure 5:**
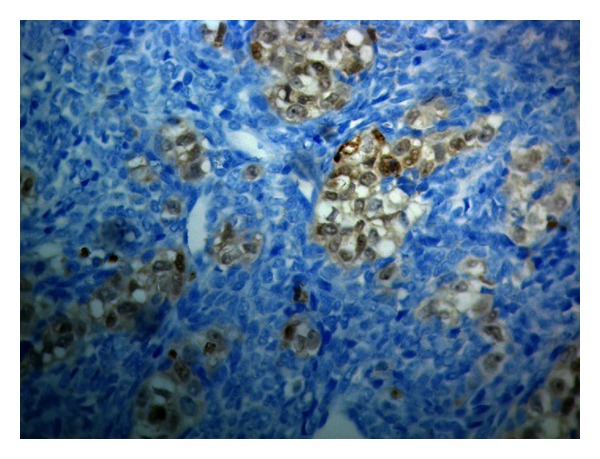
Groups of tumor cells show positive nuclear and cytoplasmic immunostaining for calretinin (DAB, Haematoxylin ×400).

The surgical specimen weighted 106 gr and consisted of the testis measuring 6.6 × 6 × 4 cm, the epididymis 2.5 in length, and the spermatic cord measuring 9.2 × 2.5 × 2.5 cm. On cross-sections, the testis presented a whitish, solid tumor measuring 4.2 × 2 × 3.1 cm. Histology showed a sex-cord tumor of the testis (Figure 2). The tumor was formed of medium-size, spindle-shaped, or epitheliod cells with indistinct cell borders and clear cytoplasm. The growth pattern varied in different areas of the tumor, being diffuse, insular, trabecular, and in places with formation of microfollicular structures containing eosinophilic material, the characteristic Call-Exner bodies (Figure 3). Nuclei were oval with smooth contours, and small nucleolus, frequently presenting longitudinal grooves. Mitoses were very rare. The groups of the tumor cells were separated by thin strands of fibrous stroma, showing hyalinization in some areas. No invasive growth, necrosis or vascular invasion was seen. Immunohistochemistry followed in order to prove the diagnosis of GST, using an automated immunoperoxidase method (Nexes, Ventana, USA). The immunoprofile of the tumor was CD99 positive (Figure 4), calretinin positive (Figure 5), inhibin positive, alpha smooth muscle actin positive, vimentin positive, estrogen receptor negative, progesterone receptor negative, keratin AE1/AE3 negative, alpha fetoprotein (AFP) negative, CD117 (KIT) negative, and placental alkaline phosphatase negative. The remaining testicular parenchyma presented focal atrophy and hyalinization of the spermatic tubules. The tunica albuginea, the epididymis, and the spermatic cord were free of tumor.

## 4. Discussion

GCTs include tumors composed of granulosa cells, theca cells, and fibroblasts in varying degrees and combinations. GCTs account for approximately 2% of all ovarian tumors and can be divided into the much more frequent adult type (95%) and the juvenile type (5%), based on histologic findings. The opposite seems to occur in males where the majority of the reported cases (50/80 cases) belong to the juvenile type of GST, while only 30 cases in the literature correspond to the adult type GST [1–10]. The juvenile type mainly concerns very young male individuals, 90% of them being less than one year of age [1–3]. This category of tumors presents with scrotal swelling, usually with no other accompanying manifestation. Juvenile GSTs in males are always benign, and simple orchiectomy suffices for cure. They may be preoperatively suspected as such, since the only other testicular that occurs in this age group is the yolk sac tumor. The latter peaks after 6 months of age and shows elevated AFP in the serum, while GSTs do not have any characteristic biochemical marker to be diagnosed of. However, AFP elevation in the serum has been reported in a few cases with juvenile GST, since this protein may be normally increased in infancy [2]. In addition, karyotypic anomalies and ambiguous genitalia have been found in approximately 20% of juvenile GSTs [3].

Adult type GSTs also manifest with scrotal swelling, even though a minority of cases has presented gynecomastia [4–10]. The age range varies greatly between 16 and 77 years, as does the size of the tumors, which may be from less than 1 cm up to 13 cm in diameter. Diagnosis depends on histology, where the characteristic Call-Exner bodies along with the nuclear grooves of the tumor cells permit most of the times a straightforward diagnosis. Other patterns encountered in the female gonads such as macrofollicular, diffuse, insular, and trabecular may also be found in testicular GSTs, alone or in any combination. The number of mitoses also varies and is not considered to be of prognostic significance. Immunohistochemistry may help diagnose ambiguous cases in their differentiation from other tumor types and especially germ cell tumors. GSTs are typically positive to calretinin, CD99, and inhibin, while other markers such as smooth muscle actin may also be expressed. Most importantly keratin stains are negative, excluding embryonal carcinoma, and placental alkaline phosphatase and KIT (CD117) are also negative, excluding seminoma. AFP, which is positive in yolk sac tumor, is negative as well in GSTs. The present case was rather typical and easy to diagnose on histological grounds, even without the immunostains. Still, immunohistochemistry was also characteristic (CD99 positive, inhibin positive, calretinin positive) and established the diagnosis.

Very little is known about the histogenesis and etiology of GCTs, especially in the testes. Recently the FOXL2 gene has been implicated in the pathogenesis of the adult type GST of the ovaries. FOXL2 is a transcription factor, which plays important role in the development of normal ovaries and is a key factor in female sex determination [11]. A missense point mutation (C134W) in the FOXL2 gene at 3q22.3 chromosome is found in virtually all of the patients with adult type ovarian GST [12]. Increased expression of genes linked to cell proliferation, but decreased expression of those conferring sensitivity to cell death has been also observed [13]. One study has attributed prognostic significance to the mRNA levels expressed by the tumor [14]. In addition, 2 out of 5 male adult GSTs studied presented the same mutation of FOXL2 gene [15]. All these molecular data seem quite promising not only for the prognostic value they may offer, but also because they may open new ways of more effective targeted therapy for these tumors.

Ovarian adult GCTs are considered as potentially malignant neoplasms. Approximately 20% of these tumors will eventually recur or metastasize, even many years after the initial diagnosis [16]. In a similar fashion, approximately 20% of testicular adult GSTs have been reported to present malignant behavior [4, 8]. Hanson and Ambaye have suggested that tumor size larger than 5 cm is a feature associated with malignancy in the testes [9]. However, there are no established discriminating criteria at present in order to predict which tumors will follow an aggressive course. Sites of metastases in male cases include retroperitoneal lymph nodes (most common), liver, bones, and the lungs [4, 5, 8]. Initial management is total orchiectomy. Retroperitoneal lymphadenectomy has been additionally performed in a few cases where metastatic disease was suspected [5]. Metastatic disease may be managed with chemotherapy (etoposide alone or in combination with other agents) and adjuvant radiotherapy [5, 8]. Still, there are no specific guidelines for treatment due to the rarity of this tumor.

In conclusion, our report highlights one more case of this very rare tumor of the testis, which is quite problematic in terms of prognosis and management, and for this reason seems to have attracted the interest of many researchers recently. Long-term followup is recommended, since recurrence of the disease may appear late in the clinical course.
